# Effects of field experimental warming on wheat root distribution under conventional tillage and no‐tillage systems

**DOI:** 10.1002/ece3.3864

**Published:** 2018-01-29

**Authors:** Ruixing Hou, Zhu Ouyang, Daorui Han, Glenn V. Wilson

**Affiliations:** ^1^ Key Laboratory of Ecosystem Network Observation and Modeling Institute of Geographic Sciences and Natural Resources Research Chinese Academy of Sciences Beijing China; ^2^ Yucheng Comprehensive Experiment Station China Academy of Science Beijing China; ^3^ University of Chinese Academy of Sciences Beijing China; ^4^ National Sedimentation Laboratory USDA‐ARS Oxford MS USA

**Keywords:** bulk density, global warming, no‐tillage, soil nitrogen, root biomass

## Abstract

Despite the obvious importance of roots to agro‐ecosystem functioning, few studies have attempted to examine the effects of warming on root biomass and distribution, especially under different tillage systems. In this study, we performed a field warming experiment using infrared heaters on winter wheat, in long‐term conventional tillage and no‐tillage plots, to determine the responses of root biomass and distribution to warming. Soil monoliths were collected from three soil depths (0–10, 10–20, and 20–30 cm). Results showed that root biomass was noticeably increased under both till and no‐till tillage systems (12.1% and 12.9% in 2011, and 9.9% and 14.5% in 2013, in the two tillage systems, respectively) in the 0–30 cm depth, associated with a similar increase in shoot biomass. However, warming‐induced root biomass increases occurred in the deeper soil layers (i.e., 10–20 and 20–30 cm) in till, while the increase in no‐till was focused in the surface layer (0–10 cm). Differences in the warming‐induced increases in root biomass between till and no‐till were positively correlated with the differences in soil total nitrogen (*R* ^2^ = .863, *p *<* *.001) and soil bulk density (*R* ^2^ = .853, *p *<* *.001). Knowledge of the distribution of wheat root in response to warming should help manage nutrient application and cycling of soil C‐N pools under anticipated climate change conditions.

## 1 INTRODUCTION

The growth and distribution of roots have a critical impact on the nutrient and water uptake by crops, and consequently on crop growth and yield (Kuchenbuch, Gerke, & Buczko, 2009; Kuzyakov & Blagodatskaya, 2015; Wuertz et al., 2006). As a result of anthropogenic buildup of greenhouse gases, the global surface temperature is predicted to increase by 3.7°C by 2100 (IPCC, 2013). Elevated temperatures have been found to directly influence wheat's growth, development, and grain yield, by changing photosynthesis and phenology development (Cai et al., 2015; Gessner, Arndt, Tiedemann, Bartel, & Kirschbaum, 2006; Hou, Ouyang, Li, Wilson, & Li, 2012). As the aboveground growth and development of wheat are both tightly related to its belowground growth (Qin, Niklas, Qi, Xiong, & Li, 2012), it is expected that the form and distribution of roots will be affected when wheat is exposed to increased environmental temperatures.

Till and no‐till tillage systems (henceforth till and no‐till), as the two major tillage systems around the world, are also widely used in China. Compared with till, no‐till has the advantage of maintaining soil fertility because of limited soil disturbance; however, the increasing soil surface bulk density associated with no‐till may result in the decline in crop yield in some region, compared to that with till (Christopher, Lal, & Mishra, 2009). Previous studies have found that the distribution of wheat roots strongly depends on the tillage system, with more roots being distributed on the surface soil layer under no‐till than under till (Qin, Stamp, & Richner, 2004). Different soil properties (such as moisture, bulk density, and N‐fertilizer application position) have been reported to contribute to root stratification under no‐till relative to till (Muñoz‐Romero, Benítez‐Vega, López‐Bellido, Fontán, & López‐Bellido, 2010; Officer, Dunbabin, Armstrong, Norton, & Kearney, 2009; Plaza‐Bonilla, Álvaro‐Fuentes, Hansen, Lampurlanés, & Cantero‐Martínez, 2014). Under warming, higher temperature has been reported to strongly affect soil properties, such as soil moisture or N cycling (Hou, Ouyang, Wilson, Li, & Li, 2014; Rustad et al., 2001). As such, there is a need to examine the warming‐induced changes in the root biomass and distribution, especially under the different tillage systems.

Higher temperature has been reported to strongly affect plant biomass by changing plant photosynthesis and stimulating compensatory growth (Wan, Xia, Liu, & Niu, 2009; Zhao, Chen, & Lin, 2008). Warming affects plant and root growth by decreasing soil moisture (θ) and enhancing soil nitrogen (N) availability by stimulating soil organic matter (SOM) decomposition (Rustad et al., 2001). Niu and Wan (2008) attribute the reduction in photosynthesis under warming to soil drying, which in turn suppresses carbon (C) allocation to the roots. Studies have found that warming increased moisture in the deeper soil layers, which resulted in deeper root growth and increased biomass in an alpine meadow (Xu, Luo, Shi, Zhou, & Li, 2014). Furthermore, considering the important role of soil water on the delivery of nutrients to the roots, warming‐induced higher transpiration is expected to affect root nutrient uptake (Inselsbacher & Näsholm, 2012). Previous studies have reported contrasting findings on wheat shoot biomass, with increases or decreases in aboveground biomass observed under warming relative to that under the ambient temperature (Batts, Morison, Ellis, Hadley, & Wheeler, 1997; Hou, Ouyang, Li, Wilson, et al., 2012; Ottman, Kimball, White, & Wall, 2012; Patil, Lægdsmand, Olesen, & Porter, 2010; Tian et al., 2012). Thus, there are huge uncertainties in the responses of root growth and production to the warming‐induced changes in soil properties.

Along with the varied responses of root biomass to higher temperatures, root distribution also could be affected by warming, specifically depending on tillage systems. Firstly, soil moisture is expected to be better maintained under no‐till than in till, because of the undisturbed surface soil residue cover in the former, which has been found to partly counterbalance warming‐induced soil drought and higher temperature (Davin, Seneviratne, Ciais, Olioso, & Wang, 2014; Hou et al., 2014). Less influence of warming on soil moisture and temperature under no‐till than under till has been reported in the North China Plain (Hou et al., 2014). Secondly, the greater soil bulk density (BD) in the surface soil layer under no‐till, acting as a barrier, is also expected to decrease the distribution of the warming‐induced changes in the root biomass to the deeper soil layers. Such knowledge is needed to better manage the availability of nutrients to crops and water use under changing climates.

As the third major crop in global production, wheat has principle contribution to human diets and the global demand for wheat is increasing (Shewry & Hey, 2015). Wheat is grown widely, mainly in the tropic and subtropics. As a result, numerous studies have reported the importance of wheat in world food production, and the significant responses of its growth and production to higher temperature (Lobell & Ortiz‐Monasterio, 2007; Mo, Liu, Lin, & Guo, 2009; Piao et al., 2010). As such, clarifying the responses of the biomass and distribution of wheat root to warming under the two main tillage systems, till and no‐till, would be helpful to estimate the threats to food security in the warmer future. Here, we hypothesized that: (1) warming increases winter wheat root biomass, (2) the increase is greater under no‐till than under till, and (3) in the shallow layer the redistribution of roots within the soil profile depends upon the tillage system.

## 2 MATERIALS AND METHODS

### 2.1 Study area

This study was conducted in the field plots of a long‐term (since 2003) conservation tillage experiment located in the North China Plain (NCP, 36°50′ N, 116°34′ E, elevation: 20 m a.s.l.). Before the treatment of 2003, the study field was a fruit garden. The setup of the field experiment is described in detail in a previous study (Hou, Ouyang, Li, Tyler, et al., 2012; Hou, Ouyang, Li, Wilson, et al., 2012). Briefly, the site is located in a temperate semiarid climate, with mean annual temperature of 13.6°C and mean precipitation of 575 mm from 1985 to 2014. Approximately 70% of annual precipitation occurs between June and September. The soil is classified as Calcaric Fluvisol according to the World References Base for soil resources (WRB, 2014). Soil texture (0–30 cm) is silty loam (sand, 12%; silt, 66%; clay, 22%), with a pH of 7.1. Winter wheat (*Triticum aestivum* L.) and summer maize (*Zea mays* L.) were double cropped according to a common practice in the NCP.

Winter wheat was irrigated on 5 May 2011 and 15 May 2013 (40–50 mm each time), respectively. In the plots that were tilled, after the harvest of the maize crop, standing crop stubble of each treatment was cut to approximately 10 cm, and all other residues were removed. A rotary tiller was used with a tillage depth of about 10–15 cm, which fully incorporated standing stubble into the soil before winter wheat planting. In the no‐till treatment, maize residues were chopped into pieces (about 5 cm length) by hand and retained on the soil surface. The residue mass retained for no‐till was about 10 Mg ha^−1^ year^−1^ with 4 Mg ha^−1^ year^−1^ of wheat and 6 Mg ha^−1^ year^−1^ of maize.

The total N application rate for no‐till and till treatments was 285 kg N ha^−1^ year^−1^ for wheat. The base fertilizer, along with phosphorus (P) and potassium (K), was applied as a compound inorganic chemical fertilizer containing N (as urea), P (as P_2_O_5_), and K (as K_2_O) at a ratio of 12:19:13 and with application rates of 116 kg/ha of N, 178 kg/ha of P, and 122 kg/ha of K as the base fertilizer for the crop in both tillage systems each year. Considering residue N (50 kg/ha of N), the inorganic N input was 66 kg/ha of N for no‐till. For topdressed N, during the re‐greening stage, the remaining 169 kg ha^−1^ year^−1^ of N was applied as urea for both till and no‐till systems. The base fertilizer application of all treatments was the same as for topdressing: October 6 and March 3. All other management procedures were identical for the two systems with spraying of herbicide (2,4‐D butylate) and insecticide (40% dimethoate) in May.

### 2.2 The experimental design

**Figure 1:**
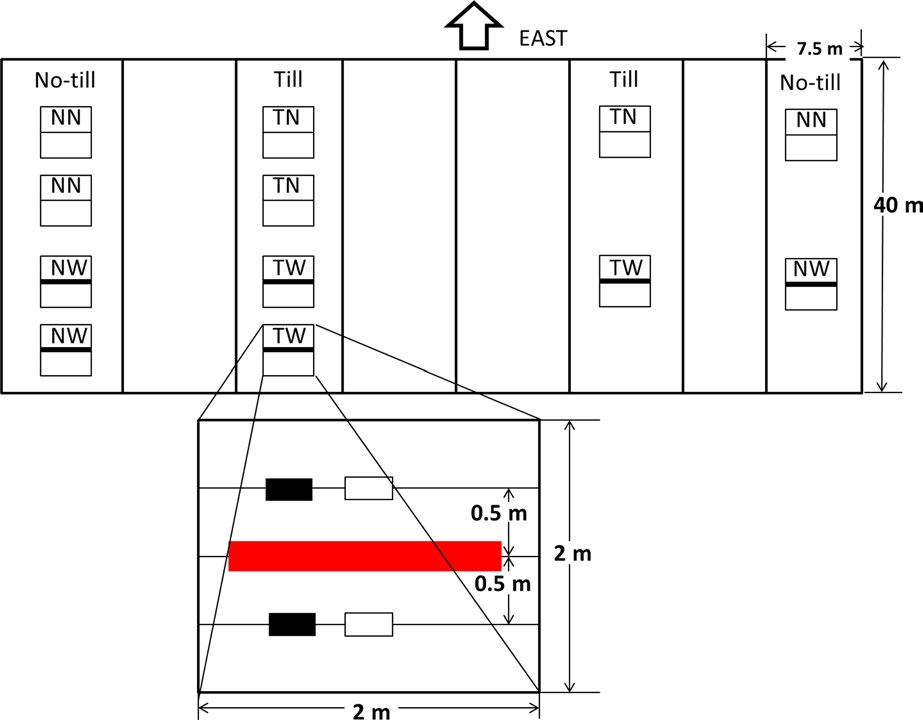
Layout of the warming experiment design. The thin line indicated the position of the “dummy” infrared heater for control plots while the thick line indicated the position of the real infrared heater in warmed plots. In the cutout, the red rectangular area was the infrared heater, the solid rectangle, and open rectangle indicated locations of thermocouples and moisture sensors, respectively. TN, till with no‐warming; TW, till with warming; NN, no‐till with no‐warming; NW, no‐till with warming

Winter wheat was exposed to two temperature regimes (warmed and nonwarmed) since February 2010. The study included four treatments: tilled with warming and nonwarming (TW and TN); no‐till with warming and nonwarming (NW and NN), respectively, each treatment was 2 m × 2 m, and had three replicates. To wheat, each plot has nine row of wheat which has a 15‐cm space between two rows. To maize, each plot has three rows with a 60‐cm space between two rows. The warmed plots were continuously heated using MSR‐2420 infrared heater (Kalglo Electronics Inc., Bethlehem, PA, USA). The heater was placed 3 m aboveground and nonwarmed plots also had a “dummy” heater 3 m aboveground. Soil temperature (at 5, 15, and 25 cm depth) and θ (at 5, 15, and 25 cm depth) were monitored by PT100 thermocouples and FDS100 soil moisture sensors (Unism Technologies Incorporated, Beijing). The thermocouples and moisture sensors were arranged symmetrically and vertically to the infrared heater with 1 m distance between the pair in each plot and connected to a datalogger. Temperature and moisture measurements were taken every hour. Details of the setup and instrumentation are showed in Figure 1. The average radiation of the infrared heater of 92 W/m^2^. Detailed measurements and calculation were described in Hou, Ouyang, Li, Tyler, et al. (2012); Hou, Ouyang, Li, Wilson, et al. (2012).

### 2.3 Sampling and measurements

Wheat root and aboveground (shoot) biomass were sampled when wheat was at maturity (i.e., harvest time) on 4 and 13 of June in 2011, 5 and 12 of June in 2013 for warmed and nonwarmed plots, respectively. To reduce the impact of root sampling between years, we separated each 2 m × 2 m plot into a pair of 2 m × 1 m subplots. Roots were sampled in one subplot of each plot for 2011, and the other was used for 2013. Wheat root distributions and soil properties were determined on three‐dimensional (3D) monoliths (Kuchenbuch et al., 2009). Each soil monolith was 40 cm long (perpendicular to wheat rows, *x*‐direction), which included two rows of wheat, 10 cm wide (parallel to wheat rows, *y*‐direction) and 30 cm deep (*z*‐direction). Each monolith was subdivided into 12 cube‐shaped 1,000 cm^−3^ samples. Three replicate monoliths were taken for each of the four treatments; thus, there were 144 cubic samples in total. Between samples, the distance was 7 m at least. Shoots were collected on each monolith.

Roots were extracted from each cubic sample by placing the sample on a sieve with mesh size of 1.0 mm and manually washing soil off the roots using a nozzle under low water pressure. Root and shoot weights were determined following oven drying at 80°C for 48 hr. Soil samples nearby each root samplings were collected for soil total N and BD after root sampling. Soil total N was quantified using a Kjeldahl digestion procedure with NH_4_‐N analyzed colorimetrically (Gallaher, Weldon, & Boswell, 1976). BD was obtained from oven dry mass relative to the sample volume.

### 2.4 Statistical analysis

We analyzed the differences between warming effects on till and no‐till root biomass (ΔRM’) and the differences on soil properties (soil temperature, θ, soil total N, and bulk density) between TW (ΔRM_till_) and NW (ΔRM_no‐till_).

(1) $$\Delta {\text{RM}}^{\prime }=\Delta {\text{RM}}_{\mathrm{till}}-\Delta {\text{RM}}_{\mathrm{no}-\mathrm{till}}$$

We used three‐way ANOVA to examine the effects of tillage, warming, and year on soil water content and temperature for each soil layer (0–10, 10–20, and 20–30 cm), shoot and total root (0–30 cm) biomass. Differences were considered significant at *p *<* *.05. Means of main effects were compared using the least significant difference test after a significant ANOVA test. Pearson's linear correlations between the parameters were also performed with SPSS. All significant differences were considered at *p *<* *.05 level. All statistical analyses were conducted with SPSS software (SPSS for Windows, version 11.5, SPSS Inc., Champaign, IL).

## 3 RESULTS

### 3.1 Effects of warming on soil temperature and moisture

**Figure 2:**
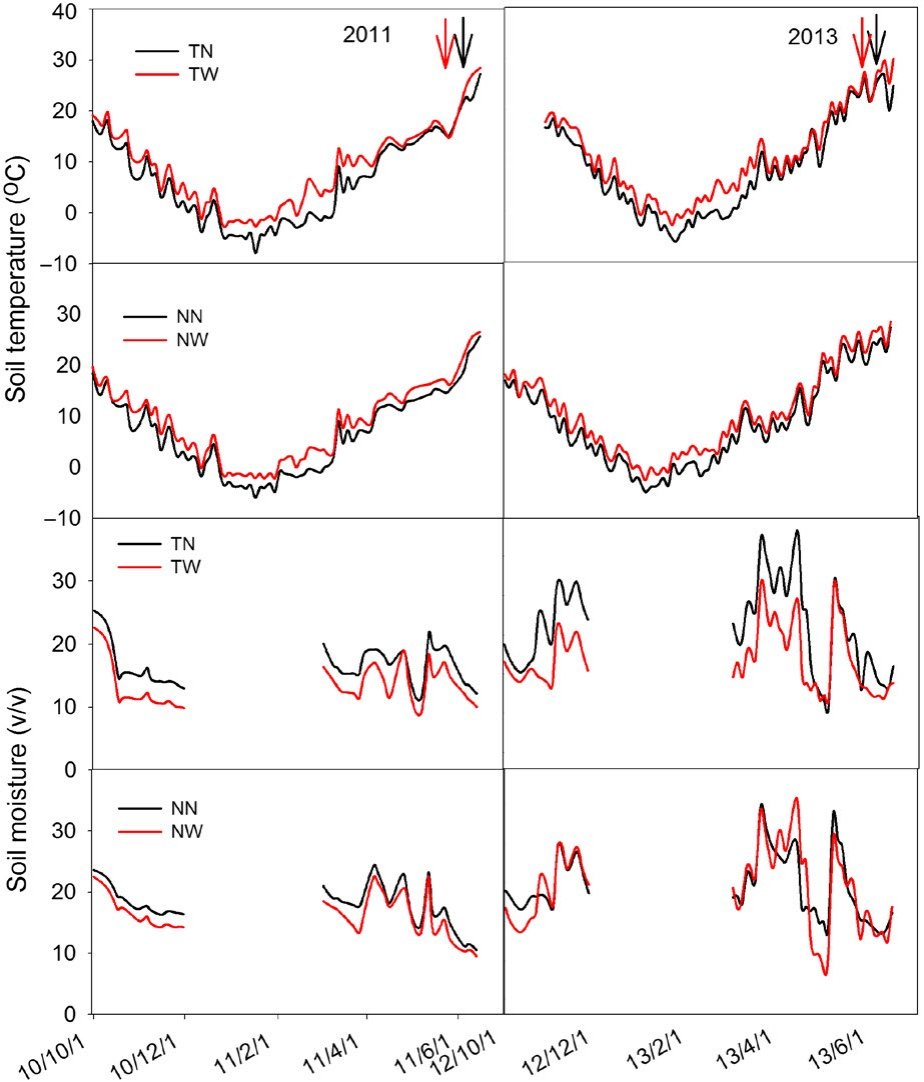
Daily mean soil temperature and water content for the four treatments (TW and TN stand for conventional tillage with warming and without warming, respectively; NW and NN stand for no‐tillage with or without warming, respectively). For soil temperature is from October 2010 to June 2011, October 2012 to June 2013. Soil water was not recorded from December to February for frozen each year. Warmed plots (TW and NW) are in red lines and control plots in black lines. The red and black arrows were the sampling times for warmed and control, respectively

Warming treatments significantly increased soil temperature and decreased θ (Figure 2 and Table 1). The increased soil temperatures ranged from 2.13 to 0.38°C, with a declining increase with soil depth. Also, the increased soil temperatures were significantly higher in till than in no‐till, and the mean difference in the increase between till and no‐till was 0.4°C (Table 1). Despite periodic irrigation of wheat, warming significantly (*p *<* *.05) decreased the soil volumetric water content across the three soil depths under both till and no‐till. Similar to that in soil temperature, the largest decline in θ was observed in the surface soil layer (0–10 cm) (Table 1). A significant interaction effect of warming and tillage on θ was found only in the 0–10 cm layer (*p *=* *.042) (Table 2).

**Table 1:** Changes in the mean soil temperature (*T*) and soil moisture (θ) under the no‐till (NT) and till (T) systems due to warming treatments

Treatments	NT	T
Soil *T* (°C) (0–10 cm)	1.66 ± 0.18	2.13 ± 0.31
Soil *T* (°C) (10–20 cm)	0.72 ± 0.06	1.05 ± 0.11
Soil *T* (°C) (20–30 cm)	0.38 ± 0. 06	0.74 ± 0.08
θ (0–10 cm)	−1.51 ± 0.24	−1.84 ± 0.21
θ (10–20 cm)	−1.07 ± 0.18	−1.26 ± 0.16
θ (20–30 cm)	−0.56 ± 0.11	−1.06 ± 0.17

All *p *<* *.05. The minus sign indicates a decrease. Soil *T* values were measured from 1 January 2011 to 15 June in 2011 and 2013. Soil moisture values were measured from 1 March to 15 June in 2011 and 2013.

**Table 2:** Results (*p* values) of three‐way ANOVA on the effects of warming (W), tillage system (T), year (Y), and their interactions on shoot biomass, total root biomass (0–30 cm) (Root_total_), soil temperature (ST), and soil moisture (θ) of three soil depths (0–10, 10–20, and 20–30 cm). All data were from 2011 and 2013 wheat seasons

Source of variance	ST			θ			Shoot_mass_	Root_total_
	0–10	10–20	20–30	0–10	10–20	20–30		
W	b	a	a	b	b	a	b	b
T	—	—	—	a	—	—	b	—
Y	b	a	a	a	b	b	b	b
W × T	—	—	—	a	—	—	—	—
W × Y	—	—	—	—	—	—	—	—
T × Y	—	—	—	—	—	—	b	—
W × T × Y	—	—	—	—	—	—	—	—

—, no significance.

a Significant at the .05 level.

b Significant at the .001 level.

No‐till had a greater BD than till in the 0–10 cm soil layer in 2011 and 2013, under both the nonwarmed (NN vs. TN) and warmed (NW vs. TW) treatments (Table 3). The differences in BD between the tillage treatments were greater under nonwarming than warming (NN vs. TN was greater than NW vs. TW). However, the differences in BD between the tillage systems and between the warming treatments decreased with depth.

**Table 3:** Soil bulk density under four treatments in 2011 and 2013

	Depth (cm)	TN	TW	NN	NW
2011	0–10	1.41 (0.04)b	1.40 (0.04)b	1.53 (0.02)a	1.50 (0.03)a
	10–20	1.43 (0.05)b	1.43 (0.04)b	1.56 (0.04)a	1.49 (0.05)a
	20–30	1.47 (0.03)a	1.49 (0.03)a	1.44 (0.06)a	1.43 (0.02)a
2013	0–10	1.34 (0.10)b	1.36 (0.06)b	1.51 (0.06)a	1.48 (0.07)a
	10–20	1.51 (0.09)a	1.48 (0.17)a	1.44 (0.12)a	1.42 (0.13)a
	20–30	1.39 (0.13)a	1.40 (0.08)a	1.46 (0.11)a	1.45 (0.09)a

TN, till with no‐warming; TW, till with warming; NN, no‐till with no‐warming; NW, no‐till with warming. Values are means with the standard deviation in parenthesis (*n* = 3); values within a row followed by different lowercase letters are significantly different (*p *<* *.05).

The soil total nitrogen (STN) distribution was affected by the tillage and warming treatments, and STN decreased with depth (Table 4). There were significant differences between till and no‐till in the 0–10 cm soil layer with respect to STN, and STN was greater under NN than TN by 12.2% in 2011 and by 21.7% in 2013, while it was greater under NW than TW by 7.7% in 2011 and by 19.0% in 2013. Till tended to have higher STN than no‐till in the deeper (10–20 and 20–30 cm) soil layers, and in 2013 the warmed treatments tended to have higher STN than the nonwarmed treatments in the deeper (10–20 and 20–30 cm) layers.

**Table 4:** Soil total nitrogen under four treatments in 2011 and 2013

	Depth (cm)	TN	TW	NN	NW
2011	0–10	1.14 (0.03)b	1.17 (0.04)b	1.28 (0.02)a	1.29 (0.04)a
	10–20	0.93 (0.02)a	0.91 (0.03)a	0.85 (0.02)b	0.84 (0.02)b
	20–30	0.83 (0.06)a	0.80 (0.03)a	0.71 (0.05)b	0.72 (0.04)b
2013	0–10	1.15 (0.03)b	1.21 (0.05)b	1.27 (0.07)a	1.34 (0.12)a
	10–20	0.98 (0.10)ab	1.04 (0.08)a	0.91 (0.03)b	0.95 (0.09)ab
	20–30	0.82 (0.08)a	0.88 (0.03)a	0.80 (0.04)a	0.84 (0.06)a

TN, till with no‐warming; TW, till with warming; NN, no‐till with no‐warming; NW, no‐till with warming. Values are means with the standard deviation in parenthesis (*n* = 3); values within a row followed by different lowercase letters are significantly different (*p *<* *.05).

### 3.2 Biomass of roots and shoots

**Figure 3:**
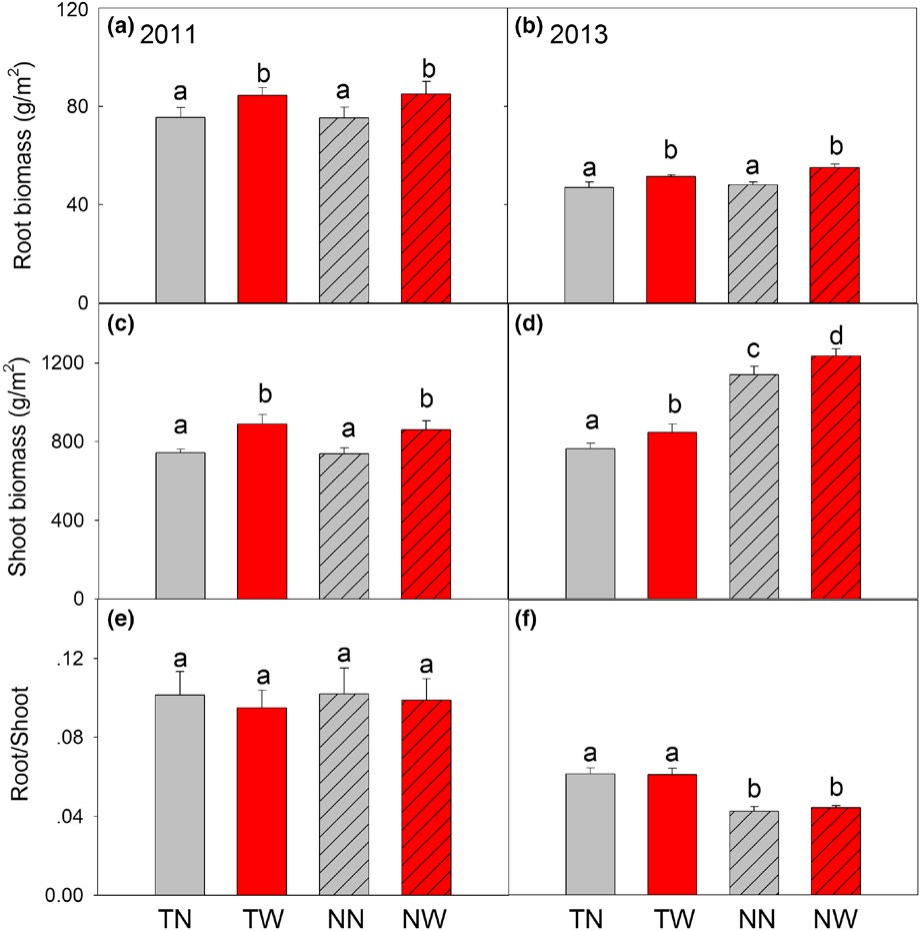
Root (a, b), shoot (c, d) and root/shoot ratio (e, f) under four treatments in 2011 and 2013. Different letters indicate significant (*p *<* *.05) difference among four treatments at same soil layer. TN, till with no‐warming; TW, till with warming; NN, no‐till with no‐warming; NW, no‐till with warming

Warming significantly increased the total wheat root biomass (0–30 cm) under both the till and no‐till systems (Figure 3). During 2011 and 2013, warming increased root biomass by 12.1% and 9.9% under till, and by 12.9% and 14.5% under no‐till. Warming also significantly increased the wheat shoot biomass (Figure 3), by 19.8% and 11.6% under till and 10.9% and 16.8% under no‐till, in 2011 and 2013, respectively. Given similar increases, there was no effect of warming on the root/shoot ratio under till and no‐till in both the years. Between the two tillage systems, wheat shoots were significantly higher under no‐till than under till in both the warmed and nonwarmed plots, in 2013.

### 3.3 Distribution of roots

**Figure 4:**
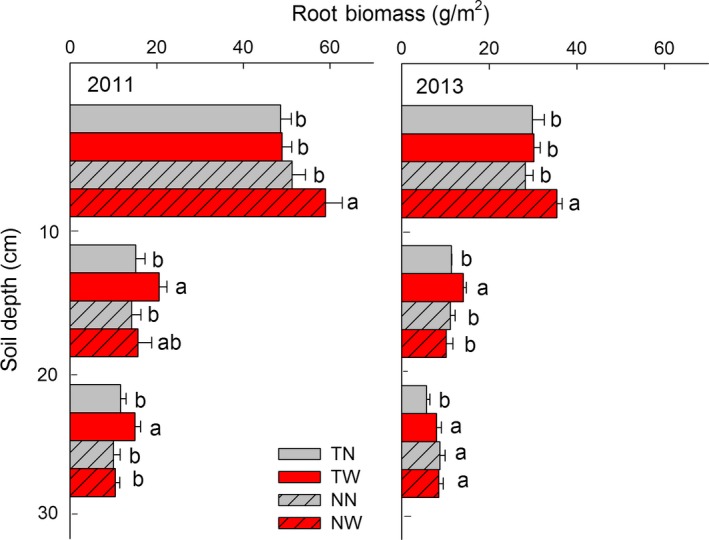
Root biomass distribution at 0–10, 10–20, and 20–30 cm soil layers in 2011 and 2013. Different letters mean significant (*p *<* *.05) differences among the four treatments at same soil layer. TN, till with no‐warming; TW, till with warming; NN, no‐till with no‐warming; NW, no‐till with warming

**Figure 5:**
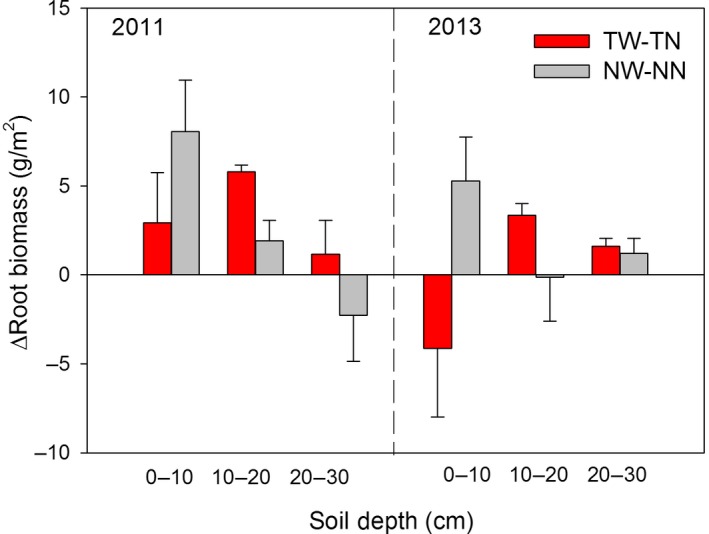
Effects of warming on root biomass of three soil depths in 2011 and 2013. *TW‐TN* warming effects on root biomass for till; *NW‐NN* warming effects on root biomass for no‐till

The effect of warming on the root biomass distribution within the soil profile depended on the tillage system and soil depth (Figure 4). Under the till system, positive effects were observed on root biomass distribution in both the 10–20 and 20–30 cm layers, but no consistent differences were observed in the surface layer (0–10 cm) in 2011 and 2013. In contrast, the no‐till system exhibited only positive effects on root biomass in the surface layer (0–10 cm), with 15.3% increase in root biomass in 2011 and 25.4% increase in 2013, but no difference observed in the two subsoil layers (Figures 4 and 5). Thus, the warming‐induced root distribution in various soil depths differed between the tillage systems. We analyzed the relationships between the differences of the warming effects on the root biomass under till and no‐till (ΔRM’ = ΔRM_till_ − ΔRM_no‐till_) and the differences of the soil properties (soil temperature, θ, soil total N, and bulk density) between TW and NW. Clearly, the results showed that the differences in the root biomass distributions between till and no‐till in response to warming were significantly influenced by the differences in soil total N (*R* ^2^ = .863, *p *<* *.001) and BD (*R* ^2^ = .853, *p *<* *.001), but not affected by soil temperature and moisture (Table 5).

**Table 5:** Pearson's correlation between selected parameters

	ΔRM’	ΔST	Δθ	ΔSTN	ΔBD
ΔRM’	1.00				
ΔST	0.28	1.00			
Δθ	0.14	0.15	1		
ΔSTN	0.863^a^	0.31	−0.098	1	
ΔBD	0.853^a^	0.18	0.168	0.877^a^	1

RM, root biomass; ST, soil temperature; θ, soil moisture; BD, soil bulk density; STN, soil total nitrogen. Each parameter includes data from the three soil depths (0–10, 10–20, and 20–30 cm).

ΔRM’ = ΔRM_till_−ΔRM_no‐till_: differences between the warming effects on till and no‐till root biomass.

ΔST, Δθ, ΔSTN, and ΔBD are the differences of these parameters between till with warming (TW) and no‐till with warming (NW) treatments.

a Correlation is significant at the .01 level (2‐tailed).

## 4 DISCUSSION

### 4.1 Responses of wheat biomass to higher temperature

Higher temperature has been reported to strongly affect plant root biomass production, which depends on the balance between the negative and positive effects of increasing temperatures (Bai et al., 2010). Numerous studies have reported that plant biomass production benefits from experimental warming treatments during the entire growing season (Dormann & Woodin, 2002; Rustad et al., 2001; Wu, Dijkstra, Koch, Peñuelas, & Hungate, 2011). We observed significantly higher wheat root biomass (9.9%–14.5%) and shoot biomass (10.9%–19.8%) in the warmed plots than in the nonwarmed plots in both 2011 and 2013, which confirmed our first hypothesis, that is, warming enhanced wheat root biomass. However, warming‐induced higher temperature has been found to have negative effects on root biomass, because the elevated temperature is above the optimum physiological temperature for wheat growth (Benlloch‐Gonzalez, Bochicchio, Berger, Bramley, & Palta, 2014). Similarly, another experimental warming study with 16 planting dates spanning two and a half years has reported that elevated temperatures begin to damage wheat biomass production when the environmental temperature rises above 13.4°C (Ottman et al., 2012). Our study field was located in the North China Plain, where the annual average air temperature is only 13.1°C. Our previous study in the same field reported the mean air temperature before wheat anthesis period to be lower than 20°C (Hou, Ouyang, Li, Wilson, et al., 2012), which is far lower than the thermal limit temperature (31°C) during this stage (Porter & Semenov, 2005). Similarly, another experimental warming study in East China observed almost 40% increase in root biomass, and the authors attribute the reason for this observation to the relatively lower environmental temperature of their study area, which was located further south to our study region (Tian et al., 2014). Thus, experimental warming may have had very limited effect on the physiology of wheat growth here. Secondly, the warming‐induced drying of soil also affects the responses of plant biomass production to warming. Warming‐induced drying of soil has been found to strongly decrease root biomass in grasslands (De Boeck et al., 2007). But, another experimental warming study reports the stimulation of root biomass and shifts of roots to deeper soil with increasing temperatures (Xu & Li, 2014; Zhou, Fei, Sherry, & Luo, 2012). In our study, the warming‐induced soil θ was only from 1.51% to 1.84% (Table 1), which was relatively lower than that reported in other studies (De Boeck et al., 2007; Liu et al., 2013). We suggest that the warming‐induced soil drying had limited negative effects on wheat biomass production in our study field for three reasons. One, there was ample water supply from routine irrigation of the soil. Routine irrigation (two‐three times) during wheat growing season can strongly help in mitigating the warming‐induced soil θ decline. Also, the annual mean belowground water table in our study field remained at only 2.3 m on average from 2011 to 2016, which contributed to greater water utilization by the rhizosphere. Two, warming‐induced soil drying usually leads to an increase in the root/shoot ratio due to the differential effects of warming on the aboveground and belowground biomass production (Bai et al., 2010; Zhou et al., 2012). However, we observed similar warming‐induced increases in both root and shoot biomass, and little warming‐induced effects on the root/shoot ratios, both in 2011 or 2013. These results indicate that shoot and root growth, which define wheat growth, were still in synchrony under experimental warming. Thirdly, soil N limitation has been reported to limit the warming‐induced positive effects on plant biomass production (Wu et al., 2011). However, in our study field, the amount of chemical N‐fertilizer applied each year could supply the additional N demand of the warming‐induced increased wheat shoot biomass and root biomass. As such, we suggest that the positive responses of root biomass under the two tillage systems resulted from the very limited warming‐induced negative effect on root biomass production in our study.

### 4.2 Distribution of wheat root biomass under warming

Root distribution is vital for nutrient and water uptake to sustain crop growth, and it is significantly affected by tillage systems (Baker, Ochsner, Venterea, & Griffis, 2007). Although till and no‐till are the two main tillage systems around world, as of yet there are few studies that focus on the root distribution responses to warming under till and no‐till. In our study, we found that there was no consistent difference in the total root biomass between till and no‐till in the 2 years of study, even though there were differences in the soil properties between these two tillage systems (Figure 3a,b). The warming‐induced changes in the total root biomass between the two tillage systems were within about 10%, thereby rejecting our second hypothesis, that is, the increase is greater under no‐till than till.

In both 2011 and 2013, we observed that warming induced significantly greater root biomass distribution in the surface soil layer (0–10 cm) under no‐till, while, warming caused significantly greater root biomass distribution in the deeper soil layers (10–20 and 20–30 cm) under the till system. Thus, our third hypothesis, that the redistribution of roots within the soil profile depends upon the tillage system, was confirmed. Warming‐induced root biomass distribution could be strongly influenced by the different soil properties that affect root growth, such as soil θ, STN, and bulk density. Soil moisture availability is considered as a major limiting factor of root growth and distribution in arid and semiarid ecosystems (Zhou et al., 2012). Roots usually distribute to the deeper soil layers, where it is more wet. Studies conducted in grasslands have found that warming‐induced increased root biomasses were consistently distributed from 0 to 45 cm (Xu et al., 2014) or from 0 to 20 cm (Na, Genxu, Yan, Yongheng, & Guangsheng, 2011) soil depths with minor decreases in soil moisture. In contrast, roots were found to distribute to deeper soils upon warming‐induced soil drying (Bai et al., 2010; Zhou et al., 2012). In this study, we found that soil moisture increased with soil depth under both till and no‐till. In till, the distribution of the roots to deeper soil may be explained by the relatively higher soil moisture decline in the surface soil layer.

However, in no‐till, although soil moisture declines similar to that in till were observed, the surface distribution of roots may have been affected by the higher bulk density in the surface soil layer. BD is another important factor that affected root redistribution patterns in till and no‐till under warming. A greater bulk density under no‐tillage systems leads to crop root stratification in the soil surface layer relative to that in the tillage systems (Baker et al., 2007). Higher BD can act as a physical barrier that restricts roots from growing deeper (Qin et al., 2004). Thus, higher BD near the surface might lessen the effects of warming on the roots in the deeper soil layers and impart tillage differences in root distribution. Furthermore, distribution of STN has also been reported to affect root distribution patterns by restricting the availability of nutrients (Jackson & Schulze, 1996). A previous study has found generalized wheat root proliferation upon N‐fertilizer application to compete for nutrients in two sites, in southern Australia (Officer et al., 2009). Between the two tillage systems, the different methods of fertilizer application result in distinct N positions. In the till system, fertilizer was mixed within the plough layer (0–20 cm), which fostered greater STN in the subsoil (10–30 cm) and likely caused greater nitrogen uptake. By comparison, N‐fertilizer was applied to the surface of the no‐till system, which, therefore, resulted in greater STN in the 0–10 cm layer (Table 4). Under experimental warming, we observed a strong positive relationship between the warming‐induced changes in root biomass distribution and STN content (*p* = .863, Table 5), which indicated increased root biomass growth following the root growth pattern between till and no‐till.

### 4.3 Effects of warming‐induced root biomass distribution on the soil organic C‐ and N‐pool

Based on our findings, it appears that the differences in the warming‐induced root biomass distributions, with greater root biomass in the surface of no‐till and greater root biomass in the subsurface of till, could strongly impact the soil C and N pools of the two tillage systems. Roots are an important contributor to the soil C and N pools. Plants can allocate as much as 30%–50% of the photosynthetically fixed C to roots (Buyanovsky & Wagner, 1997). In winter wheat, up to 15% of the net C assimilation from rhizosphere occurs during the growing season (Swinnen, van Venn, & Merckx, 1995). The root‐derived N is a main source of underground N cycled among the roots, microorganisms, and soil (Kuzyakov & Xu, 2013). Additionally, warming‐induced higher root biomass indicated an increase in N‐use efficiency and less N losses relative to those in the nonwarmed plots. Thus, warming‐induced higher root biomass under both the till and no‐till systems would increase the root‐derived C and N in soil.

The output of soil C and N also could be stimulated by the distribution of wheat roots under warming. Warming positively affects root biomass by increasing root exudation and enhancing fine root turnover, both of which directly enhance SOM decomposition by a priming effect that leads to an increased CO_2_ efflux. The warming‐induced proliferation of roots in the surface soil layer (Figure 4) may intensify soil C stratification, particularly under no‐till (Hou, Ouyang, Li, Tyler, et al., 2012; Zhao et al., 2015). As a result, the stability of soil surface C may be strongly affected by mineralization under elevated temperatures (Chaplot et al., 2015; Hou et al., 2014), which threaten the soil C‐pool under the no‐till systems. However, there is little knowledge about the effects of warming‐induced wheat root distribution on soil C or N pools depending on the tillage systems, which are complex processes and are needed to estimate the agro‐ecosystem soil C stability in the face of future climate change.

## 5 CONCLUSIONS

We conclude that warming can significantly increase root biomass under both till and no‐till in irrigated wheat croplands. The distribution of warming‐induced increased root biomass was shown to depend upon the tillage system, that is, more roots were concentrated at the surface of the no‐till system, while warming increased deeper roots under the tilled system. The differences in BD and STN between till and no‐till lead to the differences in the distribution of the warming‐induced increased root biomass. Given these differential distributions, there is a need to estimate the balance between the warming‐induced C sequestration enhancement due to increased root biomass in the surface soil layer and the potential increase in the warming‐induced C decomposition, particularly in the subsurface layers under the no‐till systems. Our results could contribute to a better understanding of the C and N dynamics of croplands and manage wheat's growth and nutrient uptake in the face of global warming.

## CONFLICT OF INTEREST

None declared.

## AUTHOR CONTRIBUTIONS

Ruixing Hou, Zhu Ouyang, and Glenn V. Wilson designed the experiment. Ruixing Hou, Zhu Ouyang, and Daorui Han performed research. Ruixing Hou, Zhu Ouyang, and Glenn V. Wilson wrote the manuscript.
